# Early strong intrathecal inflammation in cerebellar type multiple system atrophy by cerebrospinal fluid cytokine/chemokine profiles: a case control study

**DOI:** 10.1186/s12974-017-0863-0

**Published:** 2017-04-24

**Authors:** Ryo Yamasaki, Hiroo Yamaguchi, Takuya Matsushita, Takayuki Fujii, Akio Hiwatashi, Jun-ichi Kira

**Affiliations:** 1 Department of Neurology, Neurological Institute, Graduate School of Medical Sciences, Kyushu University, 3-1-1 Maidashi, Higashi-Ku, Fukuoka, 812-8582 Japan; 2 Department of Clinical Radiology, Graduate School of Medical Sciences, Kyushu University, Fukuoka, Japan

**Keywords:** Multiple system atrophy, Cerebrospinal fluid, Cytokine, Interleukin-6, Monocyte chemoattractant protein-1, Magnetic resonance imaging

## Abstract

**Background:**

The pathology of multiple system atrophy cerebellar-type (MSA-C) includes glial inflammation; however, cerebrospinal fluid (CSF) inflammatory cytokine profiles have not been investigated. In this study, we determined CSF cytokine/chemokine/growth factor profiles in MSA-C and compared them with those in hereditary spinocerebellar ataxia (SCA).

**Methods:**

We collected clinical data and CSF from 20 MSA-C patients, 12 hereditary SCA patients, and 15 patients with other non-inflammatory neurological diseases (OND), and measured 27 cytokines/chemokines/growth factors using a multiplexed fluorescent bead-based immunoassay. The size of each part of the hindbrain and hot cross bun sign (HCBS) in the pons was studied by magnetic resonance imaging.

**Results:**

Granulocyte-macrophage colony-stimulating factor (GM-CSF), interleukin (IL)-6, IL-7, IL-12, and IL-13 levels were significantly higher in MSA-C and SCA compared with OND. In MSA-C, IL-5, IL-6, IL-9, IL-12, IL-13, platelet-derived growth factor-bb, macrophage inflammatory protein (MIP)-1α, and GM-CSF levels positively correlated with anteroposterior diameters of the pontine base, vermis, or medulla oblongata. By contrast, in SCA patients, IL-12 and MIP-1α showed significant negative correlations with anteroposterior diameters of the pontine base, and unlike MSA-C, there was no cytokine with a positive correlation in SCA. IL-6 was significantly higher in MSA-C patients with the lowest grade of HCBS compared with those with the highest grade. Macrophage chemoattractant protein-1 (MCP-1) had a significant negative correlation with disease duration only in MSA-C patients. Tumor necrosis factor-alpha, IL-2, IL-15, IL-4, IL-5, IL-10, and IL-8 were all significantly lower in MSA-C and SCA compared with OND, while IL-1ra, an anti-inflammatory cytokine, was elevated only in MSA-C. IL-1β and IL-8 had positive correlations with Unified Multiple System Atrophy Rating Scale part 1 and 2, respectively, in MSA-C.

**Conclusions:**

Although CSF cytokine/chemokine/growth factor profiles were similar between MSA-C and SCA, pro-inflammatory cytokines, such as IL-6, GM-CSF, and MCP-1, correlated with the disease stage in a way higher at the beginning only in MSA-C, reflecting early stronger intrathecal inflammation.

**Electronic supplementary material:**

The online version of this article (doi:10.1186/s12974-017-0863-0) contains supplementary material, which is available to authorized users.

## Background

Multiple system atrophy (MSA) is characterized by a combination of parkinsonism, cerebellar ataxia, autonomic dysfunction, and corticospinal tract impairment [1]. There are two subtypes of MSA according to the dominant clinical features: MSA-P, which presents with parkinsonism; and MSA-C, which presents with cerebellar symptoms. The cardinal features of MSA-C are common to hereditary spinocerebellar ataxia (SCA), which demonstrates variability in the age of onset and a slower progression. Considerable numbers of patients initially diagnosed with SCA later have their diagnosis altered to MSA-C [2]. Since the initial symptoms and signs of both conditions are similar, biomarkers useful for differentiating between these two diseases would be extremely useful. However, despite continuing research in this area, biomarkers for MSA-C have proven elusive.

Pathologically, MSA shows a systematic degeneration of the olivopontocerebellar, striatonigral, and autonomic nervous systems. Abundant glial cytoplasmic inclusions in the oligodendroglial cytoplasm are a pathological hallmark of this disease [3]. In addition, massive infiltration of macrophages/activated microglia in autopsied brain tissues from MSA patients suggests that glial inflammation may play a role in disease progression [4, 5]. It is reported that serum tumor necrosis factor-alpha (TNF-α) is elevated and shows an inverse correlation with MSA disease severity [6]. This observation was interpreted to suggest that inflammatory mechanisms are operating in the early stages of the disease. As glial inflammation occurs intrathecally, cerebrospinal fluid (CSF) may be more suitable for detecting inflammatory changes than peripheral blood in MSA. However, pro-inflammatory cytokines and chemokines have not been extensively studied in CSF from MSA patients.

In this study, we aimed to characterize CSF cytokine/chemokine/growth factor profiles in MSA-C and compare them with hereditary SCA to determine correlations between CSF cytokine/chemokine/growth factor profiles and disease stages, clinical severity, and brain atrophy in MSA-C.

## Methods

### Participants

All patients were examined at the Department of Neurology at Kyushu University Hospital, Japan, between 1 January 2005 and 30 June 2013. CSF samples from 32 patients with MSA-C or SCA and 15 control participants with other non-inflammatory neurological diseases (OND) were examined in the study. For diagnosis, we defined MSA-C according to the second consensus statement on the diagnosis of MSA [7]. Briefly, patients with a sporadic, progressive, adult-onset (>30 years) disease characterized by autonomic failure involving urinary incontinence, erectile dysfunction and orthostatic hypotension, poorly levodopa-responsive parkinsonism, and cerebellar syndromes including gait ataxia, dysarthria, limb ataxia, or cerebellar oculomotor dysfunction were enrolled. Patients predominantly with cerebellar ataxia were designated MSA-C. Clinical diagnosis of SCA was made on the basis of the diagnostic criteria for hereditary SCA [8]. Diagnostic guidelines for hereditary SCA comprise: (1) age at onset of symptoms >18 years; (2) predominantly progressive cerebellar ataxia with disease duration of >1 year; (3) cases with a family history of the presence of a similar disorder or with confirmed pathological genotypes, or a history of unexplained gait disturbance in first- and second-degree relatives; (4) exclusion of secondary ataxia caused by cerebrovascular disease, tumor, alcoholism, vitamin B1 or B12 deficiency, folate deficiency, drugs, neurosyphilis, multiple sclerosis, paraneoplastic cerebellar degeneration, immune-mediated cerebellitis, and hypothyroidism. We enrolled 12 hereditary SCA cases including two with SCA type 6, one with SCA type 8, two with SCA type 31, and seven with unknown mutations. The OND group included four patients with cervical spondylotic radiculomyelopathy, four with lumbar radiculopathy, four with normal pressure hydrocephalus, and one each with myopathy, cerebral vein malformation, and psychosomatic disorder. MSA patients’ clinical severity was assessed using the Unified Multiple System Atrophy Rating Scale (UMSARS) [9]. The demographic characteristics of the participants at CSF collection are summarized in Table 1. Disease duration was significantly longer in SCA patients compared with MSA-C patients, while age at disease onset did not differ significantly between the two diseases.

**Table 1:** Patients’ demographic data

	MSA-C (*n* = 20)	SCA (*n* = 12)	OND (*n* = 15)	*p* value
Age (years)	61.3	55.3	60.1	N.S.
Sex (F/M)	10/10	7/5	5/10	N.S.
Onset (years)	59.1	44.7	n/a	N.S.
Duration (months)	25.2	125.9	n/a	0.0110*

*p < 0.05. *MSA* multiple system atrophy, *n/a* not applicable, *N.S.* not significant, *OND* other non-inflammatory neurological diseases, *SCA* spinocerebellar ataxia. The *p* values were calculated using the Student *t* test

### Multiplexed fluorescent bead-based immunoassay

CSF samples were obtained from all patients by non-traumatic lumbar puncture, centrifuged within 30 min at 800 rpm (100×*g*) at 4 °C for 5 min, and the liquid phase of the CSF was stored at −80 °C until use. The levels of 27 cytokines/chemokines and growth factors in the liquid phase of the CSF, namely, interleukin (IL)-1β, IL-2, IL-4, IL-5, IL-6, IL-7, IL-9, IL-10, IL-12 (p70), IL-13, IL-15, IL-17A, interferon (IFN)-γ, TNF-α, C-X-C motif ligand (CXCL)8/IL-8, CXCL10/interferon-inducible protein-10 (IP-10), C-C motif ligand (CCL)2/macrophage chemoattractant protein-1 (MCP-1), CCL3/macrophage inflammatory protein (MIP)-1α, CCL4/MIP-1β, CCL5/RANTES, CCL11/eotaxin, granulocyte colony-stimulating factor (G-CSF), granulocyte-macrophage colony-stimulating factor (GM-CSF), platelet-derived growth factor (PDGF)-bb, basic fibroblast growth factor (FGF), vascular endothelial growth factor (VEGF), and IL-1 receptor antagonist (IL-1ra), were measured, as described previously [10, 11]. The Bio-Plex Cytokine Assay System (Bio-Rad Laboratories, Hercules, CA) was used according to the manufacturer’s instructions. The concentrations of cytokines/chemokines and growth factors were calculated by reference to a standard curve for each molecule derived from serially diluted concentrations of standards assayed in the same manner as the CSF samples. The detection limit for each molecule was determined by the recovery of the corresponding standard (calculated by: (observed concentration)/(expected concentration) × 100), and the lowest values with more than 70% recovery were set as the lower detection limits. The lower and upper detection limits and the detection rates are shown in Additional file 1: Table S1. No samples were beyond the upper detection limits. Some samples, especially in the OND samples, were below the lower detection limits.

### Magnetic resonance imaging and analysis

All magnetic resonance images (MRI) were acquired with a 1.5 T or 3 T imager (Vision or Symphony; Siemens, Erlangen, Germany; Achieva, Philips, Best, the Netherlands). Sagittal T1-weighted images were used to measure the length of the pontine base. Imaging parameters for T1-weighted images were as follows: repetition time (TR)/echo time (TE) = 400–518/10–12 m/s; field-of-view (FOV) = 240 × 267–275 mm; and acquisition matrix = 256–512 × 192. Transverse T2-weighted images were used to evaluate the hot cross bun sign (HCBS). Imaging parameters for T2-weighted images were: TR/TE = 2650–4993/80–128 m/s; FOV = 230–240 × 256–267 mm; and acquisition matrix = 256–512 × 184–373. The length of each part of the brainstem was measured: (1) vertical width of vermis; (2) horizontal width of vermis on the line through the posterior medullary velum; (3) width of pontine base; (4) horizontal width of medulla oblongata on the line through the obex (Additional file 1: Figure S1). The degrees of HCBS, which is commonly used as a disease marker for MSA-C in the pons, was classified into four grades: Grade 0, no abnormal signals; Grade 1, faint hyper-intense anteroposterior line compared with horizontal line; Grade 2, definite HCBS on a single slice; and Grade 3, prominent HCBS on two or more sequential slices [12]. These measurements/classifications were performed by a trained neuro-radiologist without knowledge of the patients’ clinical information.

### Statistical analysis

Data are presented as mean ± SEM. The Fisher’s exact probability test was used for comparisons of the detection rates of cytokines/chemokines and growth factors in each group, followed by Bonferroni’s correction for multiple comparisons. The statistical significance of the differences in CSF cytokine values among MSA-C, SCA, and OND patients was determined by one-way analysis of variance (ANOVA) with Bonferroni’s correction for multiple comparisons. For differences in cytokines with a *p* value <0.05 after correction, the Tukey test was used to determine significant differences between pairs of groups. Heatmap and clustering analyses for the correlations between each CSF cytokine were performed using JMP Pro software (ver. 12.2.0; SAS Institute, Cary, NC). Briefly, variables for each disease type were put into a multivariate platform to create correlation matrices comparing the combinations of all variables. A heatmap was created for the correlations and examined using cluster analyses. Heatmap analyses for correlations between CSF cytokine levels with disease duration, UMSARS, and MRI measurements were determined using Pearson’s correlation coefficient analysis and one-way ANOVA. Correlation analyses between CSF cytokine levels and CSF grouping were performed using Pearson’s correlation coefficient analysis and one-way ANOVA, followed by the Fisher protected least significant differentiation (PLSD) multiple comparison test. All statistical analyses were performed using JMP Pro software (ver. 11.0.0; SAS Institute Japan, Tokyo, Japan). The threshold for significance was set at *p* = 0.05.

## Results

### Comparison of CSF cytokine levels among patients with MSA-C, SCA, and OND

**Fig. 1:**
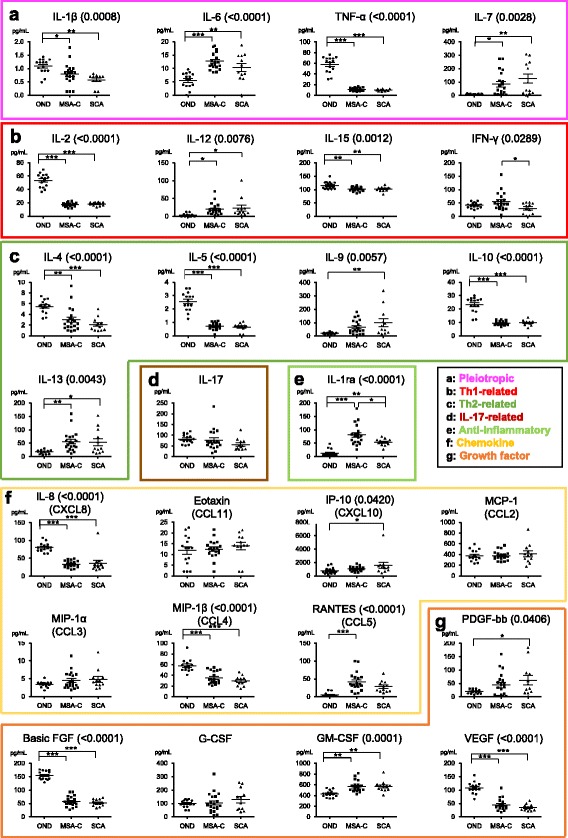
CSF cytokine levels in patients with MSA-C and SCA. **a** Pleiotropic cytokines. **b** Th1-related cytokines. **c** Th2-related cytokines. **d** IL-17-related cytokines. **e** Anti-inflammatory cytokines. **f** Chemokines. **g** Growth factors. The *p* values calculated using one-way ANOVA with Bonferroni’s correction are indicated in parenthesis next to the name of the cytokines. Post-hoc analysis was performed using the Tukey test. *p < 0.05, **p < 0.01, ***p < 0.001. CSF: cerebrospinal fluid, IL: interleukin, MSA-C: multiple system atrophy cerebellar-type; SCA: spinocerebellar ataxia, Th: type (1 or 2) T helper cell

Detection rates did not differ significantly among the groups for the 27 cytokines/chemokines and growth factors, except for IL-1ra, IL-12, and RANTES, using the Fisher exact probability test. Detection rates for IL-1ra and RANTES were significantly lower in OND patients compared with MSA-C and SCA patients (IL-1ra: *p* < 0.0001 and *p* = 0.0033, respectively, and RANTES: *p* < 0.0001 for both). The significant difference for IL-12 was absent after Bonferroni’s correction (Additional file 1: Table S1). Among the 27 cytokines measured, IL-6, IL-7, IL-12, IL-13, and GM-CSF significantly increased while basic FGF, VEGF, IL-1β, IL-2, IL-4, IL-5, IL-8, IL-10, IL-15, MIP-1β, and TNF-α decreased in both MSA-C and SCA compared with OND. IL-1ra was elevated only in MSA-C patients, while IL-9, PDGF-bb, and IP-10 increased only in SCA patients (Fig. 1; Additional file 1: Table S2).

### Cluster analyses of CSF cytokines in MSA-C and SCA patients

**Fig. 2:**
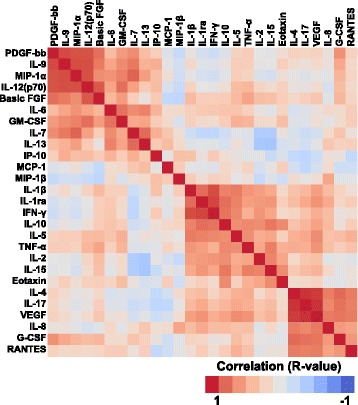
Clustering of correlations between each CSF cytokine level in MSA patients. *Color code* indicates *R* values of correlations calculated using Pearson’s correlation coefficient. CSF: cerebrospinal fluid, MSA: multiple system atrophy

Cluster analyses revealed three cytokine clusters, in which the cytokine levels in each cluster showed strong positive correlations, in MSA-C patients (Fig. 2). The first cluster comprised PDGF-bb, IL-9, MIP-1α, IL-12, basic FGF, IL-6, GM-CSF, IL-7, IL-13, and IP-10. The second cluster comprised IL-1β, IL-1ra, IFN-γ, IL-10, IL-5, TNF-α, IL-2, and IL-15. The third cluster comprised IL-4, IL-17, VEGF, IL-8, G-CSF, and RANTES. G-CSF showed positive correlations with both the first and third clusters. Two large clusters were observed in SCA patients (Additional file 1: Figure S2). The first cluster was almost the same as the first cluster observed in MSA-C patients, with strong correlations seen between each cytokine. The second and third clusters observed in samples from MSA-C patients were not detected in SCA patients. Instead, there was a large second cluster in SCA patients, with weak, nonspecific correlations. Among the cytokines included in the second cluster, IL-4, IL-17, and VEGF showed relatively strong correlations similar to that seen in MSA-C patients.

### Correlations between CSF cytokine levels and clinical parameters

**Fig. 3:**
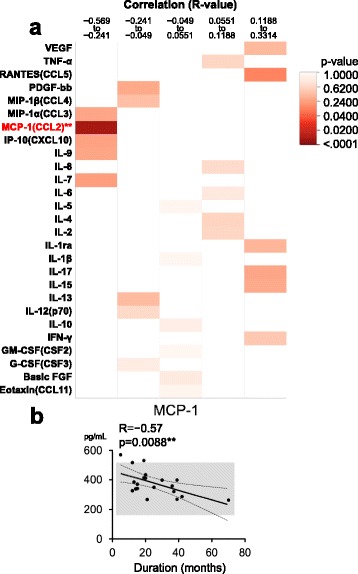
Correlations between disease durations and cytokine levels in MSA-C patients. **a** Heatmap analysis of correlations between cytokine levels and disease duration. *R* values of Pearson’s correlation coefficient analysis are divided into quintiles, and *p* values calculated using one-way ANOVA are indicated as a heatmap. Note the significant negative correlation between MCP-1 and disease duration (*R* = −0.57, *p* = 0.0088). **b** Linear regression analysis shows a strong negative correlation between CSF MCP-1 and disease duration in MSA-C. Mean value ± 2 SD observed in OND patients is indicated as a *gray bar* (mean ± 2 SD = 378.1 ± 214.4 pg/μL). CSF: cerebrospinal fluid, MCP-1: macrophage chemoattractant protein-1, MSA-C: multiple system atrophy cerebellar-type, OND: other non-inflammatory neurological diseases

**Fig. 4:**
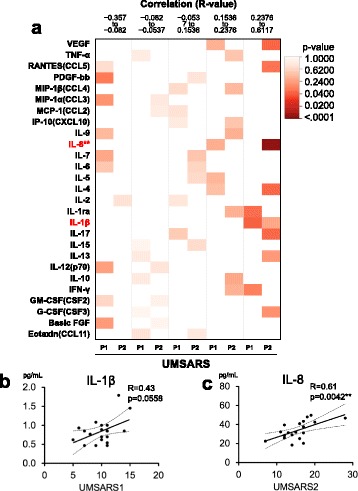
Correlations between UMSARS clinical scores and cytokine levels in MSA-C patients. **a** Heatmap analysis of correlations between cytokine levels and UMSARS part 1/part 2. *R* values of Pearson’s correlation coefficient analysis are divided into quintiles, and *p* values calculated using one-way ANOVA are indicated as a heatmap. Among the 27 cytokines studied, IL-8 and IL-1β showed correlations with UMSARS scores. **b**, **c** Linear regression analysis between IL-1β and UMSARS part 1 (*R* = 0.43, *p* = 0.0558) (**b**), and between IL-8 and UMSARS part 2 (*R* = 0.61, *p* = 0.0042) (**c**). IL: interleukin, MSA-C: multiple system atrophy cerebellar-type, UMSARS: Unified Multiple System Atrophy Rating Scale

Correlation analyses between CSF cytokines and clinical parameters in MSA-C revealed that only MCP-1 (CCL2) levels showed a significant negative correlation with disease duration (*R* = −0.57, *p* = 0.0088) (Fig. 3), although there was no significant difference in MCP-1 levels between MSA-C patients and those with either OND or SCA (Fig. 1f). The CSF MCP-1 level was up-regulated, especially at the beginning of the disease, only in MSA-C patients (Fig. 3b). No correlation with any parameter was found in SCA (Additional file 1: Figure S3). Correlation analyses between CSF cytokine levels and UMSARS part 1/part 2 showed a strong positive correlation between IL-8 (CXCL8) levels with UMSARS part 2 (motor examination scale) scores (*R* = 0.61, *p* = 0.0042), while IL-1β showed a moderately positive correlation with UMSARS part 1 scores (historical review of the functional situation) (*R* = 0.47, *p* = 0.0552) (Fig. 4).

### Correlations between CSF cytokine levels and MRI measurements

**Fig. 5:**
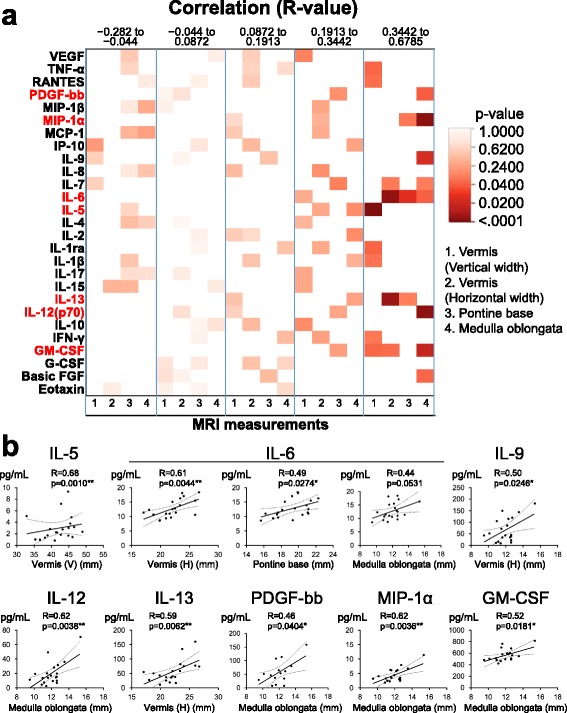
Correlations between cytokine levels and MRI measurements of each part of the hindbrain. **a** Heatmap analysis of correlations between cytokine levels and MRI measurements. *R* values of Pearson’s correlation coefficient analysis are divided into quintiles, and *p* values calculated using one-way ANOVA are indicated as a heatmap. Among the 27 cytokines studied, IL-5, IL-6, IL-9, IL-12, IL-13, PDGF-bb, MIP-1α, and GM-CSF showed significant positive correlations with MRI measurements. **b** Linear regression analysis between cytokine levels and MRI measurements for cytokines that showed significant correlations. *R* values and *p* values calculated using Pearson’s correlation coefficient analysis are indicated on each dot plot. *p < 0.05, **p < 0.01. GM-CSF: granulocyte-macrophage colony-stimulating factor, IL: interleukin, MIP: macrophage inflammatory protein, MRI: magnetic resonance imaging, PDGF: platelet-derived growth factor

Correlations between CSF cytokine levels and lengths of each part of the hindbrain (brainstem and cerebellum) were analyzed. Positive correlations between cytokines and the size of each part of the brain measured in MSA-C patients were observed (Fig. 5). Among the 27 cytokines measured, eight cytokines (IL-5, IL-6, IL-9, IL-12, IL-13, PDGF-bb, MIP-1α, and GM-CSF) showed significant positive correlations with MRI measurements. Of these, only IL-6 showed positive correlations with multiple measurements (horizontal width of vermis and anteroposterior length of pontine base and medulla oblongata). IL-5 correlated with the vertical diameter of the vermis. IL-9 and IL-13 correlated with the anteroposterior diameter of the vermis. IL-12, PDGF-bb, MIP-1α, and GM-CSF correlated with the anteroposterior diameter of the medulla oblongata. The strong positive correlations between these pro-inflammatory cytokine levels and MRI measurements were not observed in SCA patients (Additional file 1: Figure S4).

**Fig. 6:**
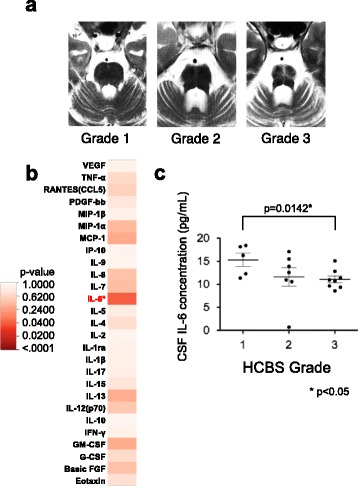
Correlations between CSF cytokine levels and HCBS scores in MSA-C patients. **a** Classification of HCBS. Grade 1, faint hyper-intense anteroposterior line compared with horizontal line; Grade 2, definite HCBS on a single slice; Grade 3, prominent HCBS on two or more sequential slices. **b** Heatmap of the *p*-values of one-way ANOVA analysis. Only the IL-6 level significantly correlated with HCBS score (*p* = 0.0453). **c** Comparison of CSF IL-6 levels with HCBS scores. The CSF IL-6 level was significantly lower in patients with higher HCBS scores using the Fisher’s PLSD multiple comparison test (*p* = 0.0142). *p < 0.05. CSF: cerebrospinal fluid, HCBS: hot cross bun sign, IL: interleukin, MSA-C: multiple system atrophy cerebellar-type, PLSD: protected least significant differentiation

We also examined whether cytokine levels correlated with the grade of HCBS (Fig. 6a) in MSA-C. There was a significant correlation between IL-6 level and HCBS grade using one-way ANOVA (*p* = 0.0453) (Fig. 6b). Fisher’s PLSD analyses showed a significantly decreased CSF IL-6 level in cases with HCBS grade 3 compared with HCBS grade 1 (Fig. 6c). In SCA patients, two cytokines, IL-12 and MIP-1α, showed significant negative correlations with MRI measurements (Additional file 1: Figure S4). Unlike MSA-C, there was no cytokine with a positive correlation in SCA.

## Discussion

The most significant finding in this study is that among the measured cytokines/chemokines/growth factors with pro-inflammatory actions, IL-6, IL-7, IL-12, IL-13, and GM-CSF were significantly elevated in CSF of both MSA-C and SCA patients when compared with OND patients. Based on cluster analyses, these factors belonged to the first cluster that was identified in both MSA-C and SCA. Interestingly, in MSA-C patients, most pro-inflammatory cytokines that showed a significant positive correlation with MRI measurements, namely the anteroposterior diameters of the pontine base, vermis, and medulla oblongata, belonged to the first cluster. These were IL-6, IL-9, IL-12, IL-13, GM-CSF, and MIP-1α and showed increased or at least unchanged CSF levels compared with OND patients. The higher these first cluster cytokine levels, the larger the size of either the pontine base, vermis, or medulla oblongata. The only exception was IL-5, which belonged to the second cluster and showed a significant decrease in CSF levels in MSA-C and SCA patients compared with OND patients, but had a significant positive correlation with the vertical diameter of the vermis. Of note is that in addition to a positive correlation with the anteroposterior diameter of the pontine base and vermis, the level of IL-6 was highest in MSA-C patients with the lowest grade of HCBS, and successively decreased as HCBS became clearer. In addition to the increased IL-6 and GM-CSF levels in MSA-C, such significant positive correlations for these first cluster pro-inflammatory cytokines with multiple MRI parameters suggest that inflammation driven by these first cluster pro-inflammatory cytokines may take place in the early stages of MSA-C when MRI changes are still minimal. These findings of increased IL-6 in CSF align with the elevation of IL-6 levels in sera from MSA-C patients in the early stage [6].

As the first cluster was similarly observed in SCA, it is possible that a similar inflammatory process may also be operating in this condition. However, such an early inflammatory process is likely to be stronger in MSA-C than SCA. Therefore, the positive correlations seen between first cluster pro-inflammatory cytokine levels and MRI measurements that relate to cerebellar and brainstem atrophy were detectable only in MSA-C and not SCA patients. Interestingly, IL-12 and MIP-1α levels negatively correlated with the size of the pontine base in SCA, indicating an increase in these cytokines in the advanced stage of disease. These findings suggest distinct inflammatory mechanisms in the progression of MSA-C and SCA.

The burden of glial cytoplasmic inclusions, a well-known pathological hallmark for MSA-C [3], is heavy at the beginning of the disease and successively decreases in the late stage [13], which parallels microglial activation [5, 13]. To the contrary, microglia numbers only slightly increased in the nucleus raphe interpositus in SCA type 3 [14, 15], although early activation of microglia was reported in a mouse model of SCA [16, 17]. These previous pathological findings align with the findings of the current study, and collectively suggest early stronger inflammation in MSA-C than in SCA.

IL-6 is released by T cells, B cells, monocytes, endothelial cells, neurons, microglia, and astroglia. In addition to infectious and autoimmune neurologic disorders, neurodegenerative diseases, such as Huntington’s disease and Parkinson’s disease, are reported to show increased CSF IL-6 levels [18, 19]. The effects of IL-6 on central nervous system (CNS) tissues are bi-directional. On the one hand, IL-6-deficient mice are more susceptible to the 1-methyl-4-phenyl-1,2,3,6-tetrahydropyridine-induced Parkinson’s disease model, suggesting neuroprotective actions of IL-6 [20]. On the other hand, IL-6-deficient mice are resistant to experimental autoimmune encephalomyelitis, an animal model of multiple sclerosis, highlighting the pro-inflammatory functions of IL-6 in CNS autoimmune disease [21]. We consider that as IL-6 behaves similarly to other first cluster pro-inflammatory cytokines/growth factors, IL-6 may exert a deleterious effect on MSA-C in the early stages together with these pro-inflammatory molecules. In this context, anti-IL-6 therapy may be effective in this intractable neurologic disease.

The second interesting finding in the current study is that only MCP-1 had a significant negative correlation with disease duration in MSA-C patients among the cytokines/chemokines/growth factors examined. CSF MCP-1 levels were greater in the early stage of MSA-C and gradually decreased over time. This is consistent with the observed higher levels of the first cluster cytokines/growth factors at the early stages of the disease. CCR2, an MCP-1 receptor, is expressed only on the surface of peripheral immune cells, mainly monocytes, functioning in the chemotaxis of such immune cells [22]. As no cells in the CNS parenchyma express CCR2, intrathecal MCP-1 solely recruits peripheral immune cells, potentiating CNS neuro-inflammation. The massive infiltration of macrophages observed in the ponto-cerebellar lesions of MSA patients could be explained by increased MCP-1 in the early course of MSA-C [4]. As IL-6 is a potent inducer of MCP-1, up-regulated IL-6 in the early stage of the disease may also facilitate monocyte/macrophage recruitment to the MSA lesions via MCP-1 production [23].

The second and third cytokine clusters were observed only in MSA-C. Among these clusters, pleiotropic pro-inflammatory cytokines such as IL-1β and TNF-α, type 1 T helper cytokines such as IL-2 and IL-15, and type 2 T helper cytokines such as IL-5 and IL-10 all decreased significantly in MSA-C patients compared with OND patients. IL-1ra, a well-known anti-inflammatory cytokine, was the only cytokine specifically up-regulated in MSA-C that also belonged to the second cluster. Except for IL-1ra, none of the cytokines in the second and third clusters showed up-regulation in MSA-C CSF. It is suggested that the second and third cytokine clusters observed only in MSA-C may reflect a host defense mechanism acting against neuro-inflammation, decreasing cytokines with pro-inflammatory actions and increasing anti-inflammatory cytokines. Intriguingly, IL-1β and IL-8 in these clusters showed positive correlations with UMSARS part 1 and part 2, respectively. It is possible that if down-regulation of the pro-inflammatory cytokine (IL-1β) and chemokine (IL-8) is insufficient, disease severity may worsen. Indeed, polymorphisms in both IL-1β and IL-8 genes were reported to confer MSA-C susceptibility, and the susceptibility allele in IL-1β is a high producer allele [24, 25]. As both IL-1β and IL-8 activate or act as a chemoattractant for macrophages and microglia, these may also potentiate macrophage/microglial inflammation in early MSA-C lesions [26, 27].

Some of the growth factors, such as VEGF and basic FGF, showed significant down-regulation in both MSA-C and SCA compared with OND. VEGF can act as a growth factor for neurons, reinforcing neuronal survival [28], while basic FGF is well known to exert strong neuroprotective functions [29]. Therefore, down-modulation of such neuroprotective growth factors may also contribute to neurodegeneration in MSA-C and SCA.

This study has some limitations. First, there were no healthy control samples because of the difficulties associated with collecting CSF from healthy people. Instead, we enrolled patients with various OND as the disease control group, as reported previously [10, 11]. By enrolling OND cases from a variety of background conditions without inflammatory mechanisms, we believe that we could reduce the risk for unexpected deviations of certain cytokine levels in CSF from the non-inflammatory disease controls. Therefore, we do not regard the higher levels of some cytokines in OND patients compared with MSA and SCA patients as evidence for active inflammation in OND patients. Second, detection rates of several cytokines were lower in OND samples compared with MSA-C and SCA samples. Therefore, we calculated the cytokine levels of these samples that were below the detection limits as the lower detection limit values. We believe that this does not significantly distort the results because the elevation rather than the reduction in cytokines in MSA-C and SCA patients compared with OND patients appears to be meaningful in terms of pro-inflammatory cytokines, and cytokines lower than the lower detection limits in SCA patients were calculated as the lower detection limits.

## Conclusions

Pro-inflammatory cytokine/chemokine/growth factor profiles in CSF appeared similar between MSA-C and SCA, suggesting involvement of neuroinflammation in both conditions. However, the pro-inflammatory cytokines/chemokines/growth factors appeared to be more clearly associated with the disease course in MSA-C rather than SCA; the earlier the disease stage in MSA-C, the higher these factors in CSF. Such a difference may be in part attributable to differences in the aggressiveness of the disease between the two conditions. The aggressive disease course in MSA-C may induce the inflammatory cytokine/chemokine/growth factor profile changes and produce a clearer cytokine profile, whereas the milder disease course in SCA may obscure such profile changes. Thus, IL-6, GM-CSF, MCP-1, IL-8, IL-12, IL-13, and IL-1β in CSF could be surrogate markers for disease course and severity in MSA-C.

With a simple comparison of CSF cytokine levels, IL-1ra, IL-6, IL-12, IL-13, and GM-CSF showed significant increases in MSA-C compared with OND. In addition, some cytokine levels are elevated in the early stages of diseases. IL-6 showed significant positive correlations with MRI measurements (vertical width of vermis, width of pontine base), and GM-CSF showed a positive correlation with the length of the medulla oblongata. An important finding of the study is that among the cytokines showing significant correlations with any of the MRI measurements, clinical scores, and HCBS scores in MSA-C, all showed higher concentrations especially in the early stage of disease. These findings highlight the contribution of the early inflammatory process in the pathogenesis of MSA-C.

## Additional file

Additional file 1: Figure S1. A schematic drawing indicating measurement of each part of the hindbrain. 1. Vertical diameter of vermis. 2. Anteroposterior diameter of vermis. 3. Anteroposterior diameter of pontine base. 4. Anteroposterior diameter of medulla oblongata. **Figure S2.** Clustering of correlations between each CSF cytokine level in SCA patients using the same order of cytokines as in MSA-C. Color codes indicate *R* values of correlations calculated using Pearson’s correlation coefficient. * *p* < 0.05, ** *p* < 0.01. CSF: cerebrospinal fluid, SCA: spinocerebellar ataxia, MSA-C: multiple system atrophy cerebellar-type. **Figure S3.** Heatmap analysis of correlations between cytokine levels and disease duration in SCA patients. *R* values of Pearson’s correlation coefficient analysis are divided into quintiles, and *p*-values calculated using one-way ANOVA are indicated as a heatmap. There were no significant correlations between cytokines and disease duration in SCA patients. SCA: spinocerebellar ataxia. **Figure S4.** Heatmap analysis of correlations between cytokine levels and MRI measurement in SCA. R-values of Pearson’s correlation coefficient analysis are divided into quintiles, and *p*-values calculated using one-way ANOVA are indicated as a heatmap. Among the 27 cytokines studied, only PDGF, IL-12(p70), GM-CSF, and MIP-1α showed significant negative correlations with MRI measurements. GM-CSF: granulocyte-macrophage colony-stimulating factor, IL: interleukin, MIP: macrophage inflammatory protein, MRI: magnetic resonance imaging, PDGF: platelet-derived growth factor, SCA: spinocerebellar ataxia. **Table S1.** Detection rates of cytokines/chemokines and growth factors in cerebrospinal fluid. **Table S2.** Summary of dysregulated cerebrospinal fluid cytokines (PDF 188 kb)

## Abbreviations

CCL: C-C motif ligand
CNS: Central nervous system
CSF: Cerebrospinal fluid
CXCL: C-X-C motif ligand
FGF: Fibroblast growth factor
FOV: Field-of-view
G-CSF: Granulocyte colony-stimulating factor
GM-CSF: Granulocyte-macrophage colony-stimulating factor
HCBS: Hot cross bun sign
IFN: Interferon
IL: Interleukin
IL-1ra: Interleukin-1 receptor antagonist
MCP-1: Macrophage chemoattractant protein-1
MIP: Macrophage inflammatory protein
MSA: Multiple system atrophy
OND: Other non-inflammatory neurological disease
PDGF: Platelet-derived growth factor
PLSD: Protected least significant differentiation
SCA: Spinocerebellar ataxia
TE: Echo time
TNF: Tumor necrosis factor
TR: Repetition time
UMSARS: Unified Multiple System Atrophy Rating Scale
VEGF: Vascular endothelial growth factor
