# Supplementary material for: Early strong intrathecal inflammation in cerebellar type multiple system atrophy by cerebrospinal fluid cytokine/chemokine profiles: a case control study
Source: J Neuroinflammation. 2017 Apr 24;14:89. doi: 10.1186/s12974-017-0863-0 (PMC5404297; doi:10.1186/s12974-017-0863-0)

Additional Fig. 1

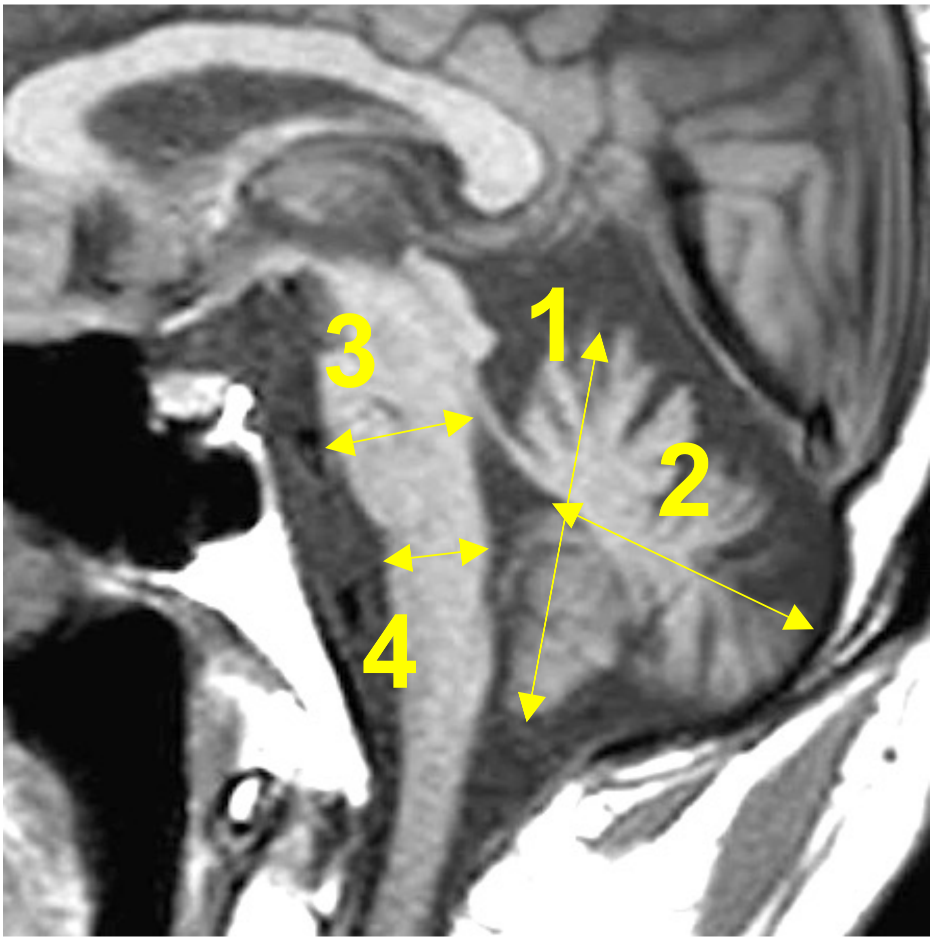

Additional Table 1 Detection rates of cytokines/chemokines/growth factors in CSF

Cytokines/chemokines/growth factors	Lower detection limit (pg/mL)	Upper detection limit (pg/mL)	Detection rate (%)			Fisher's exact probability test (corrected p-value)		
			MSA-C (n=20)	SCA (n=12)	OND (n=15)	SCA vs. OND	MSA vs. OND	MSA vs. SCA
PDGF	1.93	7901.39	15 (75)	11 (91.7)	15 (100)		ns	
IL-1β	0.14	2235.71	17 (85)	10 (83.3)	15 (100)		ns	
IL-1ra	6.4	109446.58	20 (100)	12 (100)	6 (40)		<0.0001	
						0.0033	<0.0001	ns
IL-2	0.99	15632.7	20 (100)	12 (100)	15 (100)		ns	
IL-4	0.05	3955.54	20 (100)	12 (100)	15 (100)		ns	
IL-5	0.1	1717.01	20 (100)	10 (83.3)	15 (100)		ns	
IL-6	0.09	21967.54	20 (100)	11 (91.7)	14 (93.3)		ns	
IL-7	0.15	9741.34	20 (100)	12 (100)	15 (100)		ns	
IL-8	0.15	9741.34	20 (100)	12 (100)	15 (100)		ns	
IL-9	2.1	27266.35	20 (100)	12 (100)	15 (100)		ns	
IL-10	0.13	8532.8	20 (100)	12 (100)	15 (100)		ns	
IL-12	0.14	35413.31	20 (100)	12 (100)	11 (73.3)		0.0104	
						ns	ns	ns
IL-13	0.51	8532.8	20 (100)	12 (100)	15 (100)		ns	
IL-15	6.32	6313.53	20 (100)	12 (100)	15 (100)		ns	
IL-17	2.32	28220.26	20 (100)	12 (100)	15 (100)		ns	
Eotaxin	2.02	5907.11	19 (95)	10 (83.3)	12 (80)		ns	
FGF	3.35	3444.29	20 (100)	12 (100)	15 (100)		ns	
G-CSF	1.88	28708.32	20 (100)	12 (100)	15 (100)		ns	
GM-CSF	3.45	14312.7	20 (100)	12 (100)	15 (100)		ns	
IFN-γ	5.75	32298.62	19 (95)	10 (83.3)	15 (100)		ns	
IP-10	1.9	8611.36	20 (100)	12 (100)	15 (100)		ns	
MCP-1	6.01	22755.62	20 (100)	12 (100)	15 (100)		ns	
MIP-1a	0.31	927.76	20 (100)	12 (100)	15 (100)		ns	
MIP-1b	0.24	3259.2	20 (100)	12 (100)	15 (100)		ns	
RANTES	2	6697.98	20 (100)	12 (100)	3 (20)		<0.0001	
						<0.0001	<0.0001	ns
TNF-α	1.44	5348.73	20 (100)	12 (100)	15 (100)		ns	
VEGF	0.76	12519.49	20 (100)	12 (100)	15 (100)		ns	

No samples were beyond the upper detection limits. Detection rates are expressed at the percentage of samples beyond the lower detection limit in each group. Statistically significant differences in detection rates between groups are shown as corrected p-values (p-values from the Fisher's exact probability test were corrected using the Bonferroni method). ns: not significant.

Additional Table 2 Summary of dysregulated cerebrospinal fluid cytokines

Pattern of dysregulation	Cytokines
1) Up-regulated in both SCA and MSA-C	IL-6, IL-7, IL-12, IL-13, GM-CSF
2) Down-regulated in both SCA and MSA-C	Basic FGF, VEGF, IL-1β, IL-2, IL-4, IL-5, IL-8, IL-10, IL-15, MIP-1β, TNF-α
3) Up-regulated only in SCA	IL-9, PDGF-bb, IP-10
4) Up-regulated only in MSA-C	IL-1ra
5) Unchanged	G-CSF, MCP-1, MIP-1α, RANTES, EOTAXIN, IL-17, IFN-γ

IL: interleukin, GM-CSF: granulocyte-macrophage colony-stimulating factor, FGF: fibroblast growth factor, VEGF: vascular endothelial growth factor, MIP: macrophage inflammatory protein, TNF: tumor necrosis factor, IL-1ra: interleukin-1 receptor antagonist, G-CSF: granulocyte colony-stimulating factor, PDGF-bb: platelet-derived growth factor-bb (homodimer), IP-10: interferon-inducible protein-10, IFN: interferon.

Additional Fig. 2

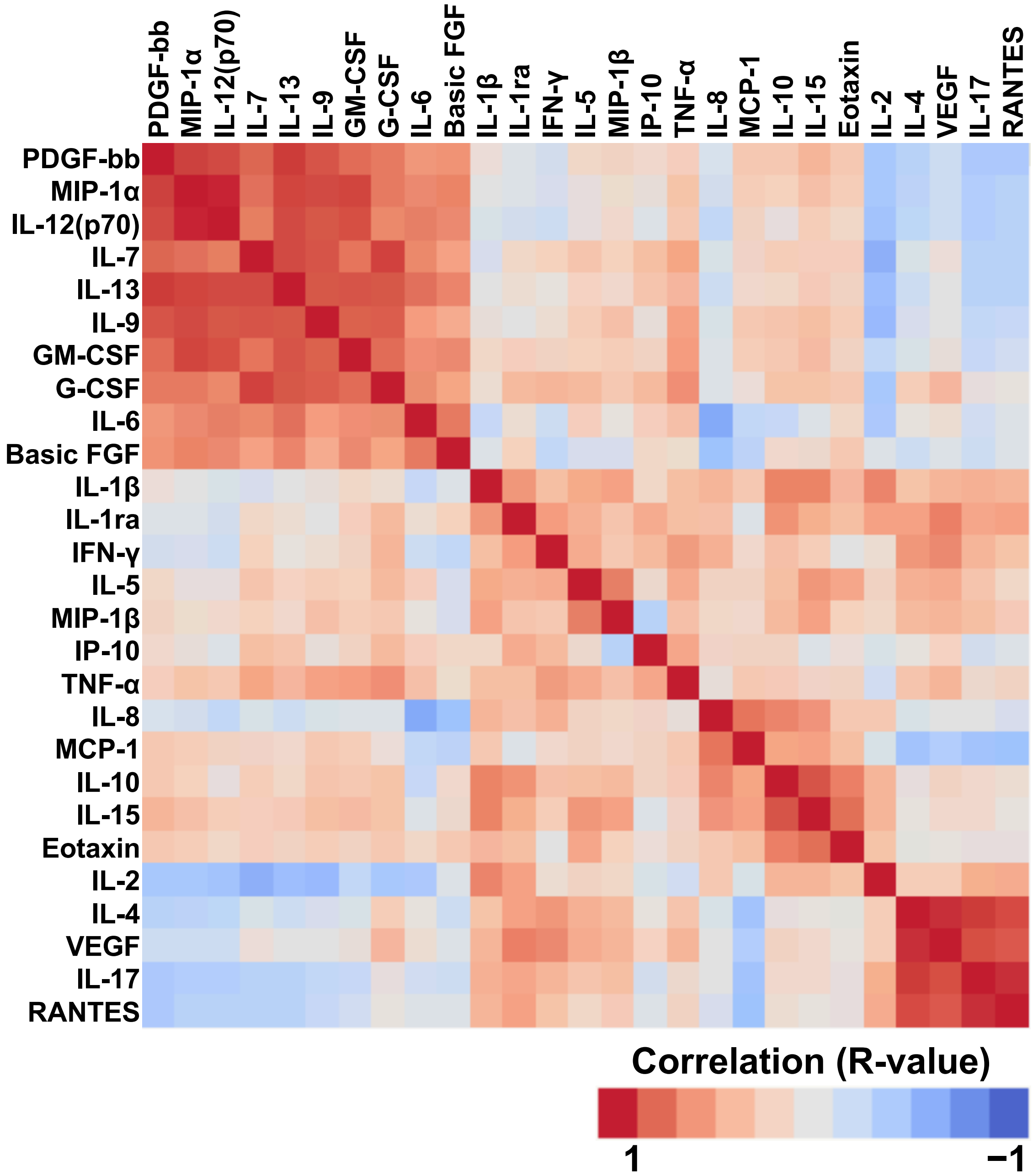

Additional Fig. 3

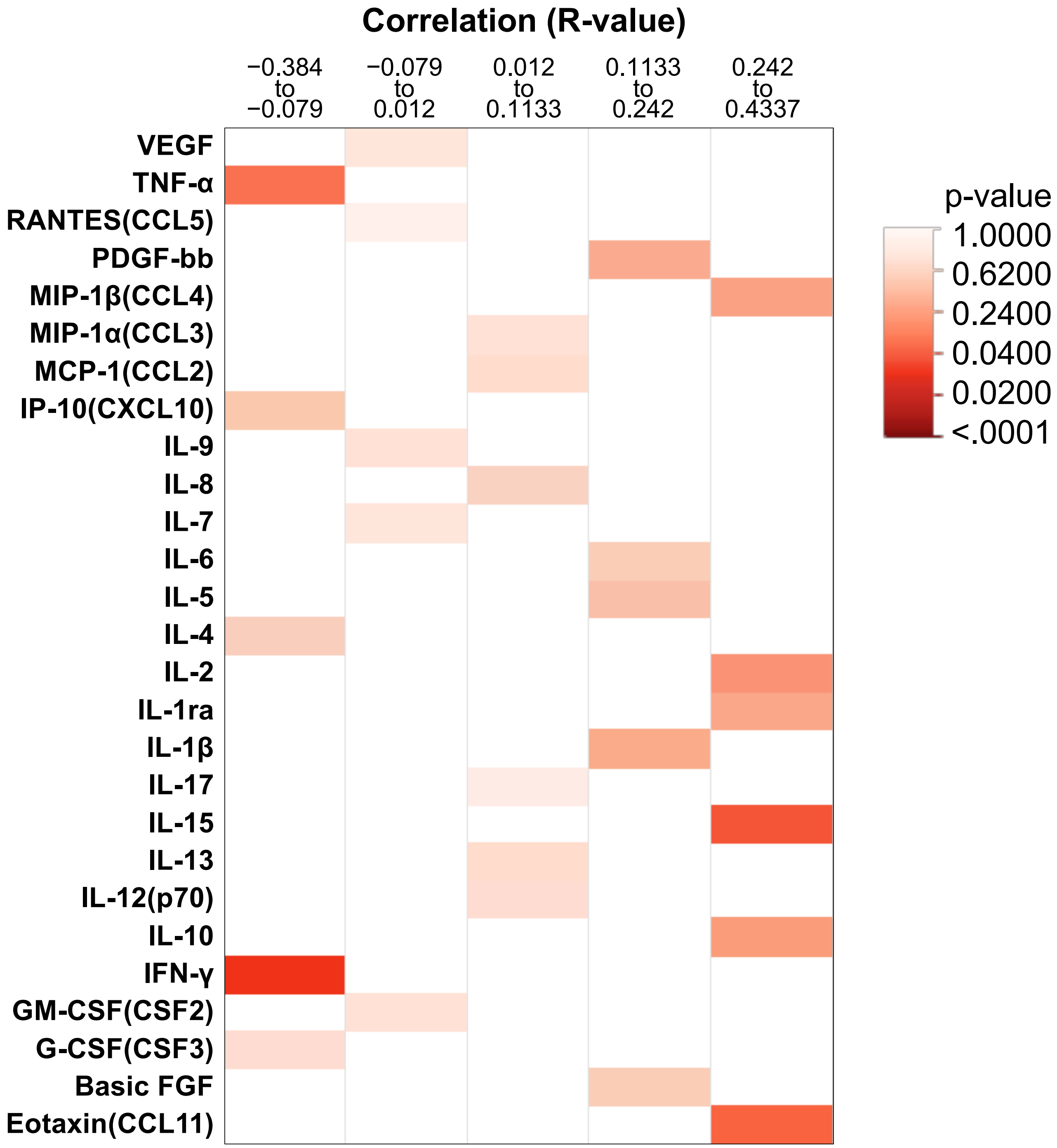

Additional Fig. 4

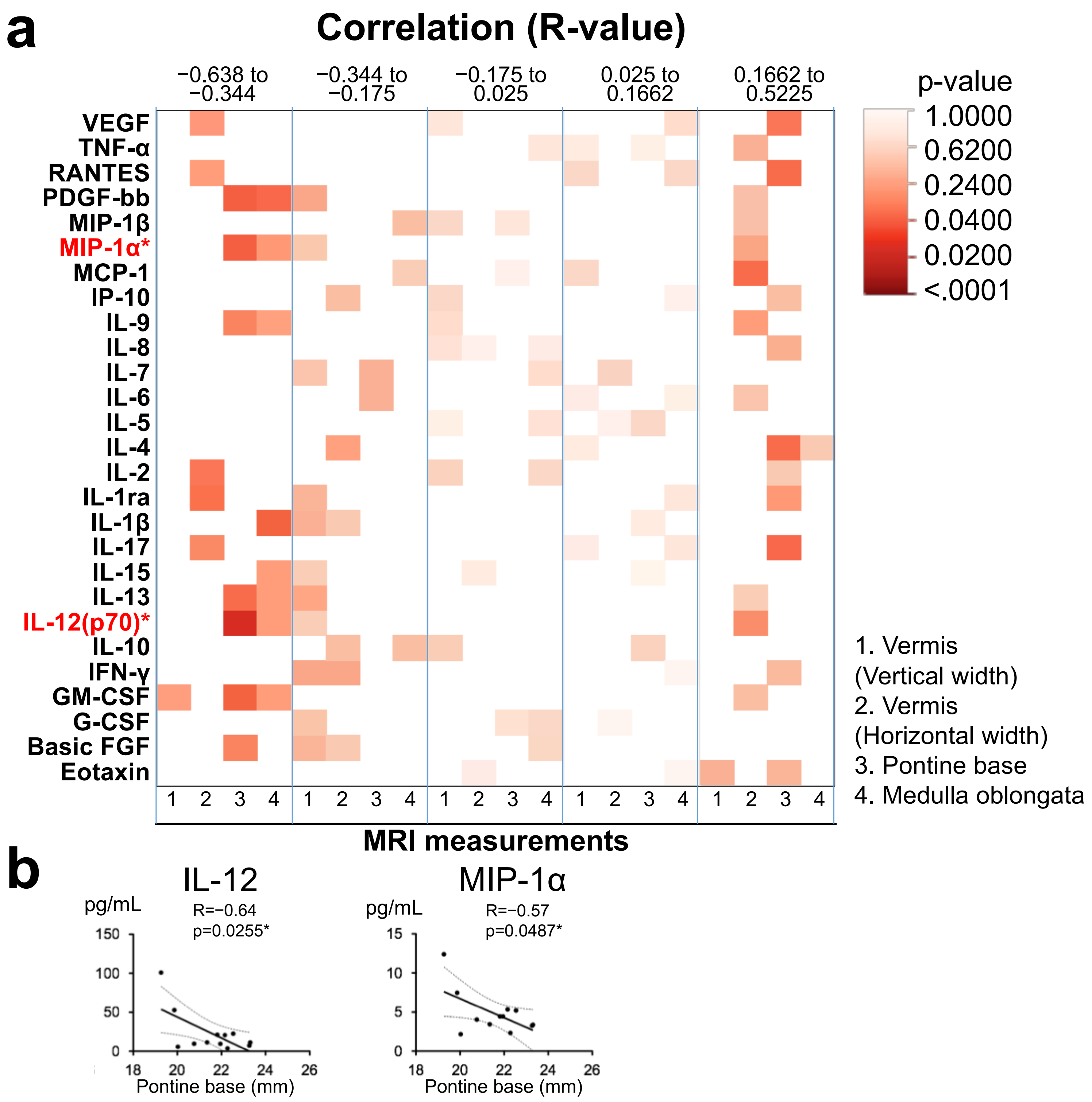

Supplement: Additional file 1: Figure S1. — A schematic drawing indicating measurement of each part of the hindbrain. 1. Vertical diameter of vermis. 2. Anteroposterior diameter of vermis. 3. Anteroposterior diameter of pontine base. 4. Anteroposterior diameter of medulla oblongata. Figure S2. Clustering of correlations between each CSF cytokine level in SCA patients using the same order of cytokines as in MSA-C. Color codes indicate R values of correlations calculated using Pearson’s correlation coefficient. * p < 0.05, ** p < 0.01. CSF: cerebrospinal fluid, SCA: spinocerebellar ataxia, MSA-C: multiple system atrophy cerebellar-type. Figure S3. Heatmap analysis of correlations between cytokine levels and disease duration in SCA patients. R values of Pearson’s correlation coefficient analysis are divided into quintiles, and p-values calculated using one-way ANOVA are indicated as a heatmap. There were no significant correlations between cytokines and disease duration in SCA patients. SCA: spinocerebellar ataxia. Figure S4. Heatmap analysis of correlations between cytokine levels and MRI measurement in SCA. R-values of Pearson’s correlation coefficient analysis are divided into quintiles, and p-values calculated using one-way ANOVA are indicated as a heatmap. Among the 27 cytokines studied, only PDGF, IL-12(p70), GM-CSF, and MIP-1α showed significant negative correlations with MRI measurements. GM-CSF: granulocyte-macrophage colony-stimulating factor, IL: interleukin, MIP: macrophage inflammatory protein, MRI: magnetic resonance imaging, PDGF: platelet-derived growth factor, SCA: spinocerebellar ataxia. Table S1. Detection rates of cytokines/chemokines and growth factors in cerebrospinal fluid. Table S2. Summary of dysregulated cerebrospinal fluid cytokines (PDF 188 kb) [file 12974_2017_863_MOESM1_ESM.pdf]
